# Efficacy of Grintuss^®^ pediatric syrup in treating cough in children: a randomized, multicenter, double blind, placebo-controlled clinical trial

**DOI:** 10.1186/1824-7288-40-56

**Published:** 2014-06-10

**Authors:** Mario Canciani, Vitalia Murgia, Davide Caimmi, Sreedhar Anapurapu, Amelia Licari, Gian Luigi Marseglia

**Affiliations:** 1 Pediatric Department, Allergology and Pulmonology Unit, School of Medicine, DPMSC, University of Udine, Udine, Italy; 2 Primary care pediatrician, scientific advisor on Phytotherapy, Italian National Health System, Mogliano Veneto, Treviso, Italy; 3 Department of Pediatrics, Immuno-Pneumo-Allergy Unit, University of Pavia, Fondazione IRCCS Policlinico San Matteo, Pavia, Italy; 4 Biometrics Department (CRO), SPRIM Advanced Life sciences (G.C.P), Milan, Italy

**Keywords:** Antitussive, Children, Cough, Efficacy, Grintuss®, Barrier effect, Safety

## Abstract

**Background:**

Cough is an extremely common problem in pediatrics, mostly triggered and perpetuated by inflammatory processes or mechanical irritation leading to viscous mucous production and increased sensitivity of the cough receptors. Protecting the mucosa might be very useful in limiting the contact with micro-organisms and irritants thus decreasing the inflammation and mucus production. Natural molecular complexes can act as a mechanical barrier limiting cough stimuli with a non pharmacological approach but with an indirect anti-inflammatory action.

**Objective:**

Aim of the study was to assess the efficacy of a medical device containing natural functional components in the treatment of cough persisting more than 7 days.

**Methods:**

In this randomized, parallel groups, double-blind vs. placebo study, children with cough persisting more than 7 days were enrolled. The clinical efficacy of the study product was assessed evaluating changes in day- and night-time cough scores after 4 and 8 days (t4 and t8) of product administration.

**Results:**

In the inter-group analysis, in the study product group compared with the placebo group, a significant difference (t4 study treatment vs. t4 placebo, p = 0.03) was observed at t4 in night-time cough score.

Considering the intra-group analysis, only the study product group registered a significant improvement from t0 to t4 in both day-time (t0 vs. t4, p = 0.04) and night-time (t0 vs. t4, p = 0.003) cough scores.

A significant difference, considering the study product, was also found in the following intra-group analyses: day-time scores at t4 vs. t8 (p =0.01) and at t0 vs. t8 (p = 0.001); night-time scores at t4 vs. t8 (p = 0.05), and at t0 vs. t8 (p = 0.005). Considering a subgroup of patients with higher cough (≥3) scores, 92.9% of them in the study product group improved at t0 vs. t4 day-time.

**Conclusions:**

Grintuss® pediatric syrup showed to possess an interesting profile of efficacy and safety in the treatment of cough persisting more than 7 days.

## Background

Cough is an extremely common problem in pediatrics [1], it is related to a poor quality of life and absence from school for children and from work for parents; moreover night-time cough is the most difficult to solve and to be tolerated. Anatomically the upper airway comprehend nasal cavities, pharynx and larynx. Upper respiratory tract infections represent the most common acute illness in the community. They can range from the self-limiting common cold, to viral and bacterial infections of pharynx and larynx and to inflammation from irritating agents. Lower respiratory infections represent a less frequent problem in children, even if a more serious one, and they involve trachea, bronchi and lungs. A persisting cough is often accompanied by upper respiratory tract infection (URTIs) [2,3], whereas other causes may be exposure to passive tobacco smoke, pollutants, aerosols and dust. A persistent cough is defined as a protracted cough lasting between 2 and 4 weeks. According to literature two out of three children aged between 0 and 4 years visit their GP at least once a year with an acute respiratory infection and up to three-quarters of these will have a cough [4]. Prospective studies of acute cough in young children in general practice have suggested that about 50% recover by 10 days and 90% by 3 weeks, so 10% of children still have problems in the third to fourth weeks [5].

In recent researches, many of the most common medications used to treat cough are nowadays studied to evaluate not only their efficacy, but also safety, in order to underline how benefits/risks ratio tent toward their beneficial effects [6-8]. In most cases, the pharmacological treatment aims to suppress the symptom (cough suppressant therapy), with synthetic agents that play an inhibitory effect on the cough reflex or act as mucolytic drugs, with the simple intent to reduce both intensity and frequency of cough in the short term, increasing the possibility to experience side effects [9-12].

Respiratory viruses, bacteria and irritants would cause a widespread inflammation of the mucosa of several anatomical areas, especially the upper respiratory tract mucosa, desquamation of the epithelial cells and damage of the nerve endings, which may then lead to a certain degree of hyper-reactivity of the cough reflex. Such a local environment may increase the sensitivity of the mucosa towards certain stimuli and local irritants, inducing a vicious circle that leads to an increased mucosal damage [13], that may facilitate irritating agents access towards the nerve endings of the sub mucosa by activating constriction and bronchial reflexes [14-16].

Starting from the above assumptions, it seems reasonable that the protective effect exerted by a mechanical barrier may represent a reliable therapeutic approach, different from the past, and able to more effectively limit the damage caused by micro-organisms and irritants on the mucosa due to an indirect anti-inflammatory action.

The barrier effect could be achieved by a particular combination of functional components (obtained from natural molecular complexes) such as resins, polysaccharides, saponins and flavonoids that are able to exert an indirect anti-inflammatory effect on the upper respiratory tract area [17-19].

In the light of these considerations, it might be interesting and innovative to investigate the efficacy of a mechanical and non-pharmacological therapeutic intervention in the treatment of persisting cough.

Aim of the present randomized, double-blind, placebo-controlled trial was therefore to assess the effectiveness of the medical device Grintuss® (the study product, classified according to the Directive 93/42/EC, containing a combination of specific fractions of substances such as resins, polysaccharides, saponins, flavonoids and sugars) in comparison with a placebo, in the treatment of cough persisting for more than 7 days.

## Methods

In the present randomized, parallel groups, double-blind vs. placebo study, 102 children aged 3 to 6 years (51 for each group) were consecutively enrolled. They were referred for persisting cough (at least 7 days up to 3 weeks, not treated with any other antitussive product) to the Pediatric Allergology and Pulmonology Unit of the Azienda Ospedaliero-Universitaria of Udine (Italy), or to the Immuno-Pneumo-Allergy Unit of the Department of Pediatrics of the University of Pavia (Italy).

Informed parental consent was obtained to be eligible for enrollment into the study. The study was performed according to the rules of Pavia and Udine Universities Ethics Committees and conducted by the CRO SPRIM ALS GCP Srl Italy.

The primary endpoint of the study was to evaluate the clinical efficacy of Grintuss® assessing the changes in the day- and night-time cough score [20] (evaluated by the specialist during the visits at t4 and t8 and recorded by parents in a daily diary) after 4 and 8 days of product administration (Table 1) [21-25]. All adverse events and severe adverse events were recorded and evaluated.

**Table 1:** Cough clinical scores (Modified from Chung 2002)

**Day-time cough score**	**Symptom**
0	Absent
1	For a short period (approximately a few minutes)
2	For 2 short periods (approximately 10 minutes)
3	Frequent cough that does not interfere with normal activities
4	Frequent cough that interferes with normal activities
5	Disturbing cough for the most part of the day
**Night-time cough score**	**Symptom**
0	Absent
1	Only at awakening/only before falling asleep
2	Awaken once/early awaken due to cough
3	Frequently awaken due to cough
4	Frequent cough for the most part of the night
5	Disturbing cough

The study product (a syrup containing specific fractions of substances such as resins, polysaccharides, saponins, flavonoids and sugars derived from *Grindelia robusta, Plantago lanceolata*, *Helichrysum italicum*, honey) was administered as it was, 4 doses a day, 5 ml each, for 8 days. The placebo was a syrup having the same organoleptic properties, viscosity and texture of the study product; it contained xanthan gum, citric acid, sugarcane, sorbate potassium (E202), acesulfame K, lemon and orange flavours, plant charcoal and beta-carotene and it was administered according to the same modalities of the study product. Both of them were supplied by Aboca S.p.A. Società Agricola, Italy. During the whole study period the following treatments were not allowed: immunostimulants or immunoregulators of biological origin, corticosteroids, antibiotics, antihistaminics and bronchodilators.

The sample size was obtained assuming a mean cough score of 3 points in both groups at baseline, and a mean change in cough score of 2 and 1 points respectively for placebo and treatment group at the end of the study, with 1.3 standard deviation.

Data from both centers participating in this study were combined. Data were summarized for demographic characteristics, efficacy observations and measurements, safety observations and evaluations using standard descriptive statistics. Safety was assessed on the frequency of adverse reactions attributable to the study product, summarized and described for each treatment group. Changes between groups in cough score were assessed with Student’s *T*-test and pooled *T*-test. Differences in efficacy between treatment and placebo groups for children with intense cough (scores ≥3) were tested by means of Fisher exact test.

## Results

All the patients (102 screened and 102 randomized to study product (n = 51) or placebo (n = 51)) were analyzed according to the intention- to-treat (ITT) population, that includes all randomized patients who took at least one dose of study product or placebo. Forty-four patients randomized to placebo and 47 patients randomized to study treatment completed the study; two patients discontinued the study (both randomized to placebo). No patients discontinued for adverse events, severe adverse events or other safety reasons.

### Demographic data and other baseline characteristics

Subjects demographics and anamnestic data are summarized in Table 2, and include age (years), gender, height (m), weight (kg), body mass index (kg/m^2^), numbers, if any, of siblings, and presence of fever. The study product group and the placebo group presented comparable demographic characteristics, without any meaningful differences as regards to all the evaluated parameters. As for the results of the cough scores, there was no significant difference in the two groups at t0.

**Table 2:** Demographic and anamnestic data from participants at the inclusion visit (t0)

		**Mean**	**Standard deviation**	**Minimum**	**Maximum**
**Study product**	Age (years)	4.90	1.00	2.9	7
n = 51	Height (m)	1.08	0.09	0.9	1.36
28 boys	Weight (kg)	19.70	4.28	11	35
	BMI	16.64	2.59	13.28	27.21
	Patients with siblings	36		1	6
	Presence of fever	2		2	2
	Cough score	2.62	1.24	2.33	1.40
**Placebo**	Age (years)	4.43	1.09	2.2	6.9
n = 51	Height (m)	1.05	0.07	0.86	1.22
26 boys	Weight (kg)	18.27	3.62	11	31
	BMI	16.29	2.36	12.17	28.11
	Patients with siblings	24		1	4
	Presence of fever	2		2	2
	Cough score	2.60	1.13	2.56	1.43

### Analysis of safety variables

A total of 34 subjects experienced an adverse event (17 in the treatment group and 17 in the placebo group) and the total number of adverse events was 55 (24 in the treatment group and 31 in the placebo group). Adverse events included mainly the appearance of fever, cold and episodes of vomiting. No severe adverse event was recorded. All adverse events were assessed as “unrelated” to the study products and the subjects recovered in all cases.

### Cough score and efficacy of the product

As regard to the primary endpoint of the study (change of cough score between t0-enrolment in the study, t4-first control visit and t8-end of the treatment), when symptoms score was compared between study product and placebo, significant differences were detected favouring the study product.

**Figure 1:**
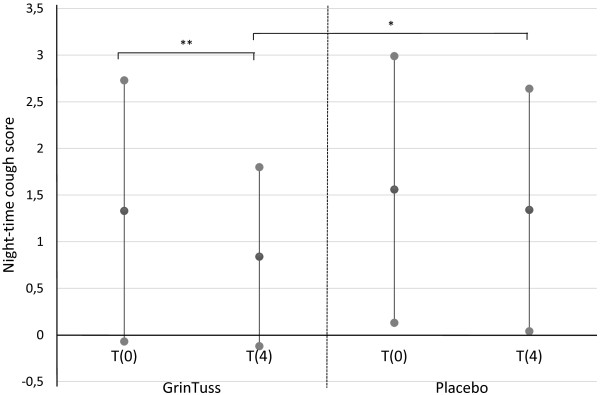
**Night-time cough score.** Night-time cough score measured at different times (day 0 and day 4) in patients enrolled for both study product and placebo groups. Means (black dots) and corresponding standard deviations show a significant decrease in mean night-time cough score for the study product group among time (*T*-test, ** *p* = 0.003)*,* and a significant difference of mean night-time cough score between study product and placebo groups at t4 (*T*-test, * *p* = 0.03).

In the inter-group analysis, in the study product group compared with the placebo group, a significant difference (t4 study treatment vs. t4 placebo, p = 0.03, *T*-test) was observed at t4 in night-time cough score (Figure 1).

**Figure 2:**
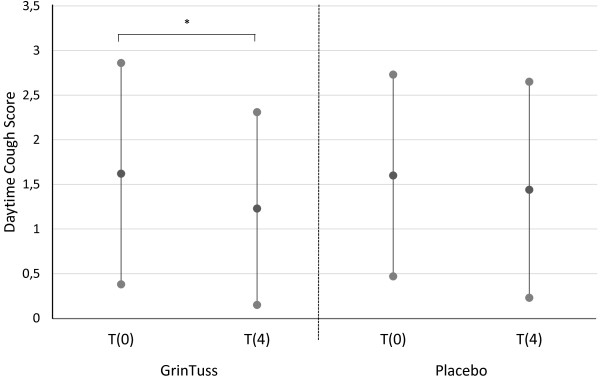
**Day-time cough score.** Day-time cough score measured at different times (day 0 and day 4) in patients enrolled for both study product and placebo groups. Means (black dots) and corresponding standard deviations show a significant decrease in mean day-time cough score for the study product group among time (*T*-test, * *p* = 0.04).

Considering the intra-group analysis, only the study product group registered a significant improvement from t0 to t4 in both day-time (t0 vs. t4, p = 0.04, *T*-test) and night-time (t0 vs. t4, p = 0.003, *T*-test) cough scores (Figures 1 and 2).

**Figure 3:**
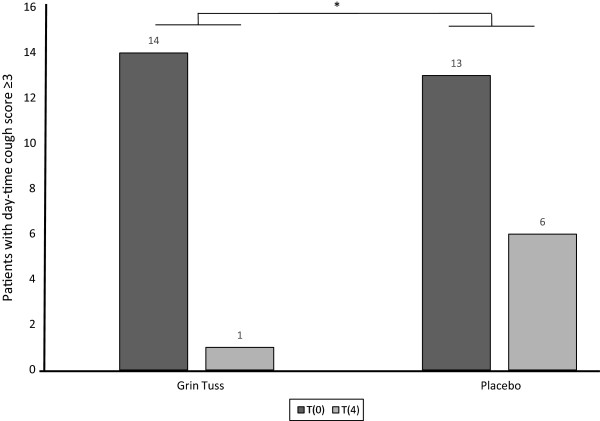
**Day-time cough score improvement in subgroups of patients with cough score ≥ 3 at t0.** It is shown the cough score in subgroups of children with score ≥ 3 in both treatment groups, between t0 and t4. As regards day-time cough in 14 children in the treatment group reporting a score ≥ 3 at t0, 13 (92.9%) improved, reporting a score < 3 at t4. As regards day-time cough in 13 children in the placebo group reporting a score ≥ 3 at t0, 7 (53.8%) improved reporting a score < 3 at t4. Fisher test shows a significant decrease of patients with day-time acute cough score between study product group and placebo group, at different times (day 0 and day 4) (* *p* = 0.03).

For the study product group, there was also a significant difference in day-time cough scores, comparing t4 vs. t8 (intra-group analysis, p =0.01, *T*-test) and t0 vs. t8 (intra-group analysis, p = 0.001, *T*-test) (data not shown). Moreover, a significant difference has been found in night-time scores comparing t4 vs. t8 (intra-group analysis, p = 0.05, *T*-test) and t0 vs. t8 (intra-group analysis, p = 0.005, *T*-test) (data not shown).In order to assess the treatment effects in children with more intense cough, evidenced by a cough score ≥ 3, a subgroup analysis was performed splitting all the participant data into two subgroups (≥3 and <3). Considering day-time cough, 13 (92.9%) out of 14 children in the study group with a t0 score ≥ 3 improved at t4, reporting a score < 3 (p = 0.03 Fisher-test) (Figure 3).

## Discussion

In children, in order to treat an episode of cough, therapies need to show a satisfactory safety profile and efficacy. Cough has a high frequency in pediatrics; it tends to persist, to generate intense discomfort in both the child and parents, and it is accompanied by the risk of unnecessary prescriptions of antibiotics and other treatments that may be associated to significant side effects [26]. Despite a certain number of studies, to date, a specific effective and safe treatment for cough has not yet been developed; such a treatment should improve both acute cough and cough persisting more than 7 days.

We then evaluated the efficacy of the study product, a medical device (Class IIa according to the definition of art. 1 of Directive 93/42/EC), whose effects can be exerted by acting as a mechanical barrier, in patients with a cough persisting for more than 7 days. It contains functional components selected for their ability to exert a barrier effect on the mucosa, and for their indirect anti-inflammatory activity. The study product is the result of a combination of specific fractions of substances such as resins, polysaccharides, saponins, flavonoids and sugars derived from *Grindelia robusta, Plantago lanceolata*, *Helichrysum italicum*, honey. Preclinical data by *in vitro* experimental assays strongly support that the study product possesses adhesive properties on human oral mucosa (under publication) and exerts a barrier effect against irritant agents even showing an indirect anti-inflammatory action.

Resins have adhesive properties, while polysaccharides have shown to have both adhesive and emollient properties [27-29]. Together, they create the mechanical barrier that limits the contact between irritants or micro-organisms and the upper respiratory tract mucosa, therefore reducing both the stimulation of nerve endings and the inflammation. Saponins are known for their ability to lower the surface tension [30]; thanks to this property, they reduce the viscosity of the mucus allowing an easier elimination. The polysaccharides attract water and moisturize the mucosa making the mucus less viscous and easier to be expelled [29]. The results of this study allow us to hypothesize that the study product can be helpful in acute cough and in cough that tends to persist even more than seven days (Figure 1). Moreover, reducing the night-time cough score at t4 (Figure 1), it is possible to achieve an improvement in quality of life, since night-time cough is less easy to control, and it creates great discomfort and causes loss of sleep both in children and parents.

The fact that the study product is effective since the first days of treatment is definitely an interesting feature, especially considering that parents, after one week of symptoms, may start to worry about it, and may seek other therapeutic intervention exposing the child to the risk of inappropriate treatment.

At last, the present study confirms the safety of the study product, since there has not been any relevant adverse event related to use of the product.

Grintuss® syrup is on the market since more than 10 years, during which the post-marketing surveillance system, in compliance with Directive 93/42/EC, has not registered incident or side effects related to the Medical Device. Grintuss® should not be used in case of known hypersensitivity to the components of medical device. No other contraindication have been registered. The present study confirms the safety of the study product.

## Conclusion

The results of this study support the evidence that Grintuss® pediatric syrup possesses an interesting profile of efficacy and safety in the treatment of cough persisting for more than 7 days. The treatment with Grintuss® is safe and effective in the relief of cough, starting on onset of application. The mechanical barrier, limiting the contact with external irritant agents or micro-organisms, helps the physiological recovery of the mucosa. The decrease of cough supports the protective role of Grintuss® thus showing an improvement in health. This effect moreover is especially evident at night.

## Competing interests

VM has been a scientific advisor and/or a speaker on educational meetings on Phytotherapy for several companies: Aboca, Dicofarm, Cristalfarma, Labomar, Lemuria, Milte. The other authors declare that they have no competing interests.

## Authors’ contributions

MC and GLM designed the study; MC, VM and GLM drafted the manuscript; DC and AL were responsible for data acquisition; SA was responsible for data management and statistical analysis. All authors read and approved the final manuscript.
